# Correlative Raman Imaging: Development and Cancer Applications

**DOI:** 10.3390/bios14070324

**Published:** 2024-06-28

**Authors:** Hossein Khadem, Maria Mangini, Somayeh Farazpour, Anna Chiara De Luca

**Affiliations:** Institute for Experimental Endocrinology and Oncology ‘G. Salvatore’, IEOS-Second Unit, National Research Council, 80131 Naples, Italy; h.khadem@ieos.cnr.it (H.K.); m.mangini@ieos.cnr.it (M.M.); s.farazpour@ieos.cnr.it (S.F.)

**Keywords:** cancer, Raman imaging, Raman spectroscopy, correlative imaging, fluorescence microscopy, atomic force microscopy, quantitative phase imaging, digital holography microscopy, mass spectroscopy imaging

## Abstract

Despite extensive research efforts, cancer continues to stand as one of the leading causes of death on a global scale. To gain profound insights into the intricate mechanisms underlying cancer onset and progression, it is imperative to possess methodologies that allow the study of cancer cells at the single-cell level, focusing on critical parameters such as cell morphology, metabolism, and molecular characteristics. These insights are essential for effectively discerning between healthy and cancerous cells and comprehending tumoral progression. Recent advancements in microscopy techniques have significantly advanced the study of cancer cells, with Raman microspectroscopy (RM) emerging as a particularly powerful tool. Indeed, RM can provide both biochemical and spatial details at the single-cell level without the need for labels or causing disruptions to cell integrity. Moreover, RM can be correlated with other microscopy techniques, creating a synergy that offers a spectrum of complementary insights into cancer cell morphology and biology. This review aims to explore the correlation between RM and other microscopy techniques such as confocal fluoresce microscopy (CFM), atomic force microscopy (AFM), digital holography microscopy (DHM), and mass spectrometry imaging (MSI). Each of these techniques has their own strengths, providing different perspectives and parameters about cancer cell features. The correlation between information from these various analysis methods is a valuable tool for physicians and researchers, aiding in the comprehension of cancer cell morphology and biology, unraveling mechanisms underlying cancer progression, and facilitating the development of early diagnosis and/or monitoring cancer progression.

## 1. Introduction

Cancer, a multifaceted disease characterized by uncontrolled cell growth, poses a significant global health challenge. Early diagnosis plays a pivotal role in combating its overwhelming impact. Detecting cancer at its emerging stage offers a window of opportunity for successful treatment and improved patient outcomes. Early intervention not only enhances the effectiveness of therapeutic measures but also diminishes the chances of cancer spreading to other parts of the body, ultimately improving patient outcomes and quality of life.

Despite extensive research efforts, cancer remains one of the leading causes of death globally, exerting a significant burden on individuals and healthcare systems worldwide. Each year, approximately 20 million new cases of cancer are reported, with roughly half of these cases, around 10 million, resulting in cancer-related deaths [1]. The complexity of cancer arises from its heterogeneity, as it encompasses a diverse array of diseases characterized by abnormal cell growth and proliferation. To gain profound insights into the intricate mechanisms underlying cancer onset and progression, methodologies that enable the study of cancer cells at the single-cell level are imperative [2,3,4]. This level of analysis allows researchers to delve deep into the molecular and cellular intricacies of cancer, uncovering the underlying genetic, epigenetic, and biochemical alterations driving tumorigenesis. By scrutinizing cancer cells at this microscopic scale, researchers can elucidate key parameters such as cell morphology, metabolism, and biochemical and molecular features [5]. Understanding these fundamental aspects of cancer cell biology is essential for deciphering tumor progression pathways and identifying potential therapeutic targets. Moreover, the ability to discriminate between healthy and cancerous cells with high precision is crucial for the development of effective diagnostic tools and targeted therapies tailored to individual patients’ needs. In this context, microscopy techniques have played a pivotal role in advancing our understanding of cancer cell biology, allowing the visualization and analysis of cellular structures and processes at unprecedented resolutions. 

Among these microscopy techniques, Raman microspectroscopy has emerged as a powerful and invaluable tool for studying cancer cells. RM utilizes the principle of Raman scattering, a phenomenon of non-elastic scattering of light by molecules, discovered by Sir C.V. Raman and K.S. Krishnan about 100 years ago [6]. They observed, in addition to elastic scattering known as Rayleigh scattering with a frequency equal to that of the radiation source, non-elastic scattering with frequencies different from that of the radiation source, which is known as Raman scattering. This phenomenon was explained in the form of the interaction of light with molecular vibrational and phonon modes [7]. Since the frequency shift between the radiation source and Raman scattering is unique for each molecular bond and its vibration type, Raman scattering is referred to as a molecular fingerprint [8]. Initially, the inherent weakness of Raman scattering signals compared to other optical scattering and emission phenomena such as Rayleigh scattering and fluorescence rendered this phenomenon not very practical. However, advancements in laser technology, filters, and optical detectors, along with the discovery of creative methods to enhance Raman signals, have led Raman spectroscopy to be recognized as a powerful analytical method for molecular analysis, surpassing its competitor, infrared spectroscopy [9]. The advantages of Raman spectroscopy include sample preparation-free, non-invasive, non-contact, label-free capabilities, and suitability for operation in aqueous environments [10]. Nonetheless, the inherent weakness of Raman scattering results in only one photon out of 10^6^ to 10^8^ incident photons undergoing Raman scattering, leading to a weak Raman signal, making detection challenging in samples with low concentrations [11,12].

The coupling of Raman spectroscopy with optical microscopy, known as Raman microscopy (RM), allows for the acquisition of both biochemical and spatial details at the single-cell level without the need for labels or disrupting cell integrity. This capability provides biochemical information about the metabolic status of cells in their natural state, enabling the differentiation between healthy and cancer cells [13,14,15,16,17]. Furthermore, by observing the Raman spectra profiles of different cellular compositions such as nucleic acids, proteins, lipids, and carbohydrates, the state of healthy/cancer cells can be evaluated and characterized to gain deeper insight into the processes governing cellular biology, cancer, and its progression [14,15,18,19,20]. Additionally, by scanning cells point-by-point, micron-scale cellular biomolecular distribution maps can be obtained, a technique known as Raman imaging [21,22,23,24]. Raman imaging can provide fundamental information about signal transduction, spatial distribution of biomolecules and drugs within cells, and temporal dynamics of intracellular changes [25,26,27]. Moreover, Raman imaging can provide insightful information for cellular assessment and examination of cell quality across various cellular life stages [28,29,30].

Raman microscopy is built around an optical microscope, making it easily combinable with other imaging techniques. This characteristic allows for a global and multifaceted view of the sample under examination. Since the optical microscope provides a detailed image of the sample’s morphology, Raman microscopy can be used to acquire specific molecular information within specific regions of the optical image. This combination of optical and Raman microscopy enables the correlation of the sample’s morphological features with its chemical properties, providing a more comprehensive and detailed view of the biological processes at play [31,32]. Furthermore, the versatility of Raman microscopy allows for easy integration with other imaging techniques, such as confocal microscopy, atomic force microscopy, and mass spectrometry, to achieve a complete and multidimensional analysis of the sample. This ability to combine different imaging techniques enables researchers to gain a deeper understanding of the biological phenomena under study and to identify meaningful correlations between various sample characteristics.

In this review, we investigate the correlation of Raman imaging with four methods, i.e., confocal fluorescence microscopy (CFM), atomic force microscopy (AFM), digital holography microscopy (DHM), and mass spectroscopy imaging (MSI). Integrating RM and CFM in correlative studies allows for a comprehensive examination of cancer cells, encompassing composition analysis, biomarker detection, and cell cycle classification, thereby providing rapid insights into cancer progression. The AFM method, besides providing cellular morphology, is capable of measuring cellular biomechanical parameters at the nanoscale. Due to the distinct adhesion and elasticity of cancer cells compared to healthy cells, AFM results exhibit a high correlation with biochemical results obtained from RM. DHM furnishes morphological information and the refractive index of subcellular components. Because of the distinct metabolism of healthy and cancer cells, organelle morphology undergoes changes that correlate with RM results. Finally, MSI is a versatile technique that can offer spatial distribution, relative content, and structural information of multiple biomolecules in biological tissues. The combination of MSI with RM extends chemical coverage, thus overcoming individual technique limitations.

The correlation of information from these various analysis methods serves as a valuable tool for physicians and researchers, aiding in the comprehension of cancer cell morphology and biology, unraveling mechanisms underlying cancer progression, and facilitating the development of early diagnosis and/or monitoring of cancer progression. Through interdisciplinary collaboration and technological advancements in microscopy, we can continue to deepen our understanding of cancer at the cellular level and translate this knowledge into improved patient outcomes in the fight against cancer.

## 2. Discussions

### 2.1. Correlative Raman Microscopy and Confocal Fluorescence Microscopy

Confocal fluorescence microscopy offers high-resolution imaging of specific molecular targets through fluorescent labeling, enabling visualization of cellular structures and protein distributions. It is a well-established and extensively utilized tool in biological research, known for its straightforward application and high-throughput capabilities. Despite these advantages, it has certain limitations. A significant issue is the potential overlap of dye spectra, which can complicate or even prevent the simultaneous analysis of multiple target molecules. Additionally, fluorescent tags can disrupt molecular transport in live cells, potentially leading to inaccurate observations. Furthermore, the dyes used in fluorescence microscopy can be cytotoxic, posing a risk of damaging the specimens being studied. Therefore, an ideal technique should be able to reveal molecular changes within the sample in a non-destructive manner, without prior knowledge of the protein or compartment to be analyzed, and without compromising the cell status. Consequently, complementary approaches, such as Raman imaging, have been proposed for this purpose. When combined with Raman imaging, these techniques provide a comprehensive view of cancer cells at the single-cell level. Indeed, CFM can offer morphological and spatial information, while Raman microscopy (RM) reveals biochemical alterations, providing a chemical fingerprint of the cell in a more physiological state for the cell. This synergistic approach not only enhances our understanding of cancer biology at the molecular level but also holds great promise for improved cancer diagnosis and treatment monitoring. There are several ways to correlate the two approaches. The most commonly used approach involves analyzing the same sample sequentially with both techniques or modifying the same microscope to acquire both imaging modalities. The first example of correlative RM and CFM was demonstrated in 2003 by Otto et al. [33]. They modified a confocal optical microscope to simultaneously provide continuous-wave two-photon-excited fluorescence microscopy and confocal Raman microscopy. They demonstrated fast image acquisition with fluorescence imaging of HeLa cells and slower, but more specific and label-free, imaging of selected areas of interest for subsequent chemical analysis with spontaneous Raman imaging. A few years later, Popp et al. presented the first characterization of unstained and fluorescence-stained blood cells using Raman spectroscopy [34]. Initially, blood cells isolated from whole blood were examined as a model system to identify the key parameters necessary for successfully combining fluorescence-labeled antibodies with Raman spectroscopy. Basically, they used fluorescent labeling to isolate some cells from the whole blood and Raman fingerprints to further define the type of blood cells. To this aim, they used a fluorescent dye with a maximum absorption at 495 nm, which is about 37 nm away from the Raman laser excitation wavelength (532 nm), allowing the acquisition of unaltered Raman spectra of fluorescence-stained blood cells isolated from whole blood without interference from fluorescence. Since the first papers, several groups conducted groundbreaking studies that utilized both RM and CFM as powerful tools in investigating cancer cells and discerning them from healthy ones [25,35,36]. By amalgamating the Raman findings with insights from fluorescence imaging, Abramczyk and colleagues deepened their understanding of the analyzed cell samples, including crucial aspects such as cell adhesion and interactions with the extracellular matrix [35]. These elements play pivotal roles in the study of cancer onset, progression, and metastasis. Indeed, while RM offers detailed biochemical information and structural insights, its limitations include lower sensitivity and the need for extensive data processing. Conversely, CFM excels in sensitivity and specificity with its fluorescent labeling, but it may suffer from photobleaching and limited depth penetration. Together, these techniques overcome individual limitations and enhance the overall understanding of complex biological systems. 

Another intriguing study, conducted by Voros et al., applied a correlative approach to examine the cell cycle of cancer cells and classify them based on their specific mitotic stages [36]. They utilized fluorescence imaging of cell DNA content as an initial screening tool to select cells for detailed examination via RM. This work is particularly interesting as it addresses the challenge of integrating images acquired with different instruments and how AI can help overcome certain traditional limitations, such as the slowness of the RM approach. Indeed, the complexity of data analysis, particularly in the reconstruction of Raman images, is a factor not to be overlooked. Only in recent years, thanks to the development of sophisticated techniques based on artificial intelligence, has significant progress been made in co-registration, spectral unmixing, and image fusion. In particular, Voros et al. developed a neural network for the analysis of complex Raman and fluorescence data to precisely categorize cells into specific classes based on their mitotic phases. In this case, the integration of these two techniques allowed the visualization of the morphology of stained molecules by CFM and correlative label-free imaging, providing information on the spatial distribution of molecular fingerprints at the subcellular level, striving to render the approach as rapid as possible and to enhance our understanding of the dynamic processes involved in cancer cell division and proliferation.

An interesting application of correlative RM and CFM is the study of the uptake and intracellular localization of nanodevices. Nowadays, nanomedicine is crucial for the development of innovative and specific treatments for diseases such as cancer. A critical aspect of preclinical studies is understanding the cellular uptake and intracellular localization of these nanodevices, both in vitro and in vivo. These aspects have traditionally been analyzed using confocal fluorescence microscopy. However, it is worth noting that fluorescence labels could potentially alter the size and chemical properties of nanovectors, thereby impacting the study of their intracellular delivery and properties. The correlation between RM and CFM has proven particularly valuable in providing a comprehensive understanding of nanodevice behavior, as demonstrated by Managò et al. [25]. In their study, they investigated the internalization and cellular localization of diatomite nanoparticles (DNPs) loaded with small-interfering RNA in a lung adenocarcinoma cell line (H3551). The internalization of the nanoparticles was examined using both RM and CFM over a period of 72 h, revealing a strong correlation between the two techniques. Of particular interest, CFM enabled rapid imaging, allowing for quick visualization of the cell status. Conversely, RM being label-free provided insight into the internal composition of the cells, revealing that DNPs were enclosed in lipid vesicles, as indicated by the co-localization of DNP Raman bands with lipid Raman signals. This combined approach not only enriches our understanding of nanodevice interactions within biological systems but also expedites the development of more effective nanomedicine-based therapies.

**Figure 1:**
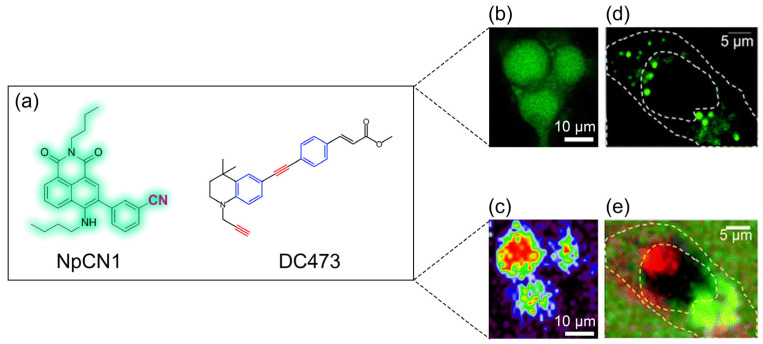
(**a**) Chemical structures of the NpCN1 and DC473 molecules. (**b**) CFM image and (**c**) Raman false-color image of 3T3-L1 cells treated with NpCN1, (**d**) CFM image, and (**e**) Raman false-color image of SW480 cells incubated with DC473. Reprinted (adapted) with permission from Ref. [37], Copyright 2021 MDPI, and from Ref. [38], Copyright 2018 Royal Society of Chemistry.

Recently, dual-tagging molecules have been demonstrated to be highly useful as they allow for the simultaneous performance of RM and CFM. Research in this field is very active, with various examples of newly developed tagging molecules found in the literature. For instance, Lin et al. developed a molecule called NpCN1, detectable by both CFM and RM due to the presence of a nitrile tag (Figure 1) [37]. The presence of the nitrile tag is particularly crucial for its use in RM. Nitrile groups have distinct vibrational frequencies that make them easily identifiable in RM, providing a clear signal without interference from the complex biological background. At the same time, these chemical tags exhibit minimal reactivity or interference with the biological system they are introduced into, thus preserving the physiological integrity of the system. NpCN1 targets lipid droplets (LDs), a well-known hallmark of cancer (Figure 1b,c). Using NpCN1, researchers obtained both biochemical information (such as the composition and state of lipids) and spatial information (such as the distribution and dynamics of lipid droplets within cells). This dual information is essential for a comprehensive understanding of lipid metabolism in the context of cancer and other diseases. The ability to use both methods on the same molecule streamlines the experimental process and provides complementary data, enhancing the overall understanding of the biological system under study.

Another interesting molecule is DC473, which serves a dual function as both a photosensitizer capable of inducing cell death and a tagging molecule detectable by both CFM and RM (Figure 1). Also, this molecule produces a strong signal in the silent zone of the Raman spectrum due to the presence of a diphenylacetylene structure [38]. In this study, DC473 was used to induce cell death in colorectal cancer cells and to study CFM, and RM was used to study its effect on the cells. DC473 was found to accumulate in LDs, detectable by both CFM and RM (Figure 1d,e). Moreover, DC473 was also found to be localized in the nuclei, but this specific subcellular localization was only detectable by RM and not by CFM (Figure 1e). The correlation between RM and CFM in this case was crucial for understanding the precise organelle localization of DC473 and elucidating its mechanism of action as a photosensitizer. Observations made with CFM were validated with RM, ensuring that the detected signals accurately represent the presence and state of DC473 within the cells.

To simultaneously acquire both fluorescence and Raman signals in a single measurement, the use of organic fluorescent dyes for specimen staining is generally avoided. This is due to the fact that their emission bands are significantly broader and more intense than Raman signals, which can obscure the Raman image. Consequently, only autofluorescence (typically weak and not very specific) can be employed [39]. In contrast, phosphorescent probes offer greater compatibility with Raman microscopy because their bands are very narrow, and their intensity is comparable to the background Raman bands [40]. By designing phosphorescent Raman tags that emit strong signals within the Raman silent region, it is possible to eliminate spectral interferences, thereby enabling more selective and accurate Raman imaging [41].

Continued advancements in the development of dual-tagging molecules and the refinement of correlative methodologies hold promise for overcoming these obstacles. As we move forward, the synergistic use of RM and CFM not only offers a deeper understanding of cancer biology but also paves the way for innovative diagnostic tools and personalized treatment strategies, marking a significant step towards more effective cancer management in the future.

### 2.2. Correlative Raman and Atomic Force Microscopy

AFM uses a nanoscale cantilever probe that interacts with a sample’s surface via extremely small forces. As the probe scans across the sample, it deflects in response to the surface topography. This deflection is measured by a laser and translated into a highly detailed 3D map, reaching nanometer-scale resolution [42,43]. Importantly, AFM not only provides topographical information but also, by carefully controlling the force of the probe, it can measure localized mechanical properties like stiffness, elasticity, and adhesion, revealing variations in these properties across cell structures and tissues [44,45,46].

The integration of Raman spectroscopy and AFM offers a uniquely detailed perspective on cancer, revealing the complex interplay between the biochemical changes and biomechanical alterations that occur as cells undergo malignant transformation. Here is a deeper dive into how this correlative approach has been leveraged in cancer studies.

#### 2.2.1. Identifying Cancer-Specific Alterations

Raman spectroscopy offers specific insights into altered metabolic pathways within cancer cells. Increased cholesterol content, a potential marker of cancer progression, can be detected through characteristic shifts in Raman peaks [47,48]. Further analysis can reveal variations in epigenetic modifications, like the levels of histone acetylation, using Raman shifts associated with methyl groups within proteins [49].AFM provides simultaneous information on the organization of the cell’s cytoskeleton, cell surface roughness, and overall elasticity—all factors known to deviate in cancer cells. Studies comparing normal and cancerous urothelial cells revealed differences in cytoskeletal organization, variations in cell surface roughness, and significantly lower elasticity and higher deformability in cancer cells [50].

**Figure 2:**
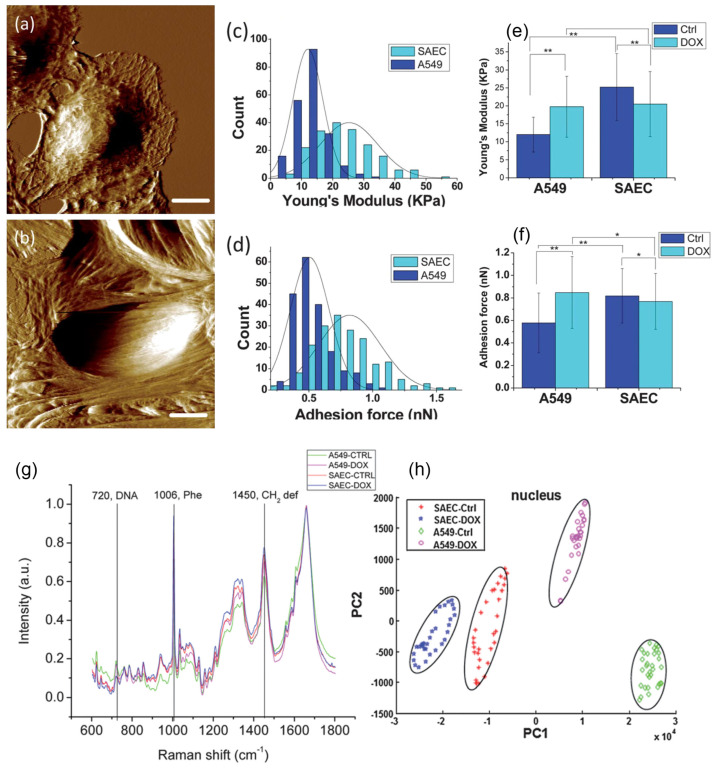
AFM images of living (**a**) human lung adenocarcinoma epithelial cell line A549 (**b**) non-cancerous human primary small airway epithelial cells (SAECs). Cells were imaged in culture media under physiological conditions. Scale bar: 10 mm. Histograms of (**c**) Young’s modulus and (**d**) adhesion force distributions of A549 cells and SAECs. Data are expressed as mean ± SD. Comparison of (**e**) Young’s modulus and (**f**) adhesion force of A549 cells and SAEC control groups and doxorubicin (70 nM, 4 h) treated groups. Values represent the mean ± SD (bar) of multiple cells. * *p* < 0.05, ** *p* < 0.01. (**g**) Average Raman spectra and (**h)** principal component analysis (PCA) of A549 cells and SAECs for the nucleus area of control and doxorubicin treatment (70 nM, 4 h) groups *(n* = 32). Reprinted (adapted) with permission from Ref. [51], Copyright 2013 Royal Society of Chemistry.

Figure 2a,b show the AFM images of human lung adenocarcinoma epithelial cell line A549 and non-cancerous human primary small airway epithelial cells (SAECs) [51]. Cancer cells exhibit distinct morphological differences from healthy cells. By calculating the Young’s modulus (a measure of stiffness) and adhesion force of the two cell lines, quantitative differences also become apparent, as shown in Figure 2c,d, respectively. As is evident, the cancer cell line A549 has a lower Young’s modulus and adhesion force compared to the healthy cell line SAEC. This can be explained by cytoskeletal changes and extracellular matrix remodeling [52,53]. After a short-term treatment with the anti-cancer drug doxorubicin (DOX) for 4 h, the biomechanical properties of A549 cells showed an increase, whereas those of SAECs decreased as shown in Figure 2e,f. This suggests that the DOX-induced response mechanisms differ between these two cell types. Raman spectral changes indicate a decrease in DNA and an increase in protein and lipid concentrations, which are attributed to DOX-induced cell apoptosis. Furthermore, principal component analysis (PCA) clearly shows that the SAEC clusters (with and without DOX treatment) are positioned close to each other, in contrast to the distinct separation between the A549 control and A549-DOX clusters. This suggests that short-term (4-h) DOX exposure has a less pronounced effect on the spectral changes in SAECs compared to A549 cells.

AFM is powerful to study the geometry and spatial distribution of nanoparticles. Plasmonic nanoparticles have the capability to enhance Raman signals in the form of surface-enhanced Raman spectroscopy (SERS). Combining the two techniques of Raman microscopy and AFM can reveal the correlation between the enhanced Raman signals originating from cancer biomarkers and the spatial positioning of plasmonic nanoparticles. This high correlation not only provides more credibility to the results of Raman spectroscopy but also enables to provide precise quantitative results of limit of detection (LoD) and Raman enhancement factor (EF) [54,55,56].

#### 2.2.2. Subcellular Analysis at High Resolution

**Figure 3:**
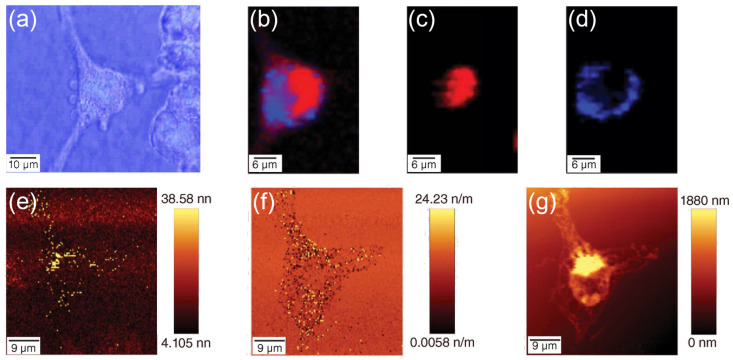
(**a**) Bright field optical microscopy image, (**b**) Raman image (red: proteins; blue: lipids), fluorescence images of (**c**) Hoechst 33342 and (**d**) Oil Red O of a living U-87 MG cell, (**e**) adhesion image, (**f**) stiffness image, and (**g**) topography image of an air-dried cell. Reprinted (adapted) with permission from Ref. [35], Copyright 2019 Future Medicine Ltd, London, UK.

Correlative Raman–AFM enables the spatial mapping of subcellular structures in unprecedented detail. Raman spectral signatures can differentiate organelles like the nucleus, mitochondria, and the endoplasmic reticulum, as well as visualize lipid droplets [1,15]. AFM imaging complements this with high-resolution topographical maps of the organelles while simultaneously probing their membrane properties [57,58]. Figure 3 shows the correlation between Raman and fluorescence images with adhesion and stiffness images obtained from AFM [35]. The accumulation of lipid droplets in the Raman and fluorescence images (blue regions) aligns very well with the adhesion image. This indicates that the lipid droplets are the most adhesive organelles in the cell. Since lipid droplet accumulation and catabolism are intricately linked to energetic metabolism, cell signaling, and are vital for cancer cell proliferation, resistance to death, and aggressiveness, these findings could be beneficial for better understanding and timely detection of cancer [59].

Studies show that fatty acid supplementation alters lipid composition and saturation levels within lipid droplets, detectable through AFM and Raman spectroscopy [58]. Moreover, AFM and Raman imaging have been applied to track variations in epigenetic markers, specifically histone and DNA methylation status within the nucleus of normal and malignant breast cancer cell lines [49].

The correlative approach can elucidate drug-induced cell death pathways. Raman spectra demonstrate a reduction in protein and DNA synthesis characteristic of apoptosis following anticancer treatment [51,60,61]. AFM measurements complement this by revealing altered cell shape, elasticity, and adhesion forces upon drug exposure [51,57,61]. These insights can help compare the efficacy of various anticancer agents. 

Specialized microfluidic devices have been developed to integrate Raman monitoring with AFM measurements [61]. This allows researchers to observe how cancer cells respond to drugs in real-time within a controlled environment, tracking changes in both chemical composition and biomechanics over the course of treatment.

Combining Raman microscopy with AFM can lead to nanometer and sub-nanometer resolutions that are much lower than the diffraction limit. In the method of tip-enhanced Raman spectroscopy (TERS), a metallic AFM tip is precisely brought into contact with the cell membrane so that it spatially coincides with the location of the laser excitation for Raman scattering [62]. Due to the surface plasmon resonance in the metallic tip, the Raman spectrum of the cell membrane, precisely beneath the tip, is enhanced. In addition to high resolution, this method also allows for operation with very low laser powers in the microwatt range, which is another advantage of this technique [63].

#### 2.2.3. Metastasis Studies and Biomarker Discovery

Studies directly comparing cancerous and metastatic cells reveal that metastatic cells often possess lower adhesion, a rounder morphology, and a softer cytoskeleton—all characteristics measurable using AFM [64,65]. Correlative Raman analysis provides complementary insights into altered lipid and nucleic acid levels associated with the increased metastatic potential [50,65].

Raman and AFM can be used to analyze extracellular vesicles (EVs), which are involved in cancer cell communication and metastasis. Size, morphology, and chemical composition of cancer-derived EVs can be determined [66,67]. Notably, Raman spectroscopy and AFM have pinpointed that cancer EVs are enriched with hyaluronic acid (HA), a potential diagnostic biomarker [66].

### 2.3. Correlative Raman and Digital Holography Microscopy

As a quantitative phase imaging technique, digital holography microscopy (DHM) revolutionizes cell study by capturing three-dimensional images of cells with unparalleled precision and depth [68]. Unlike conventional microscopy, which provides two-dimensional snapshots, DHM reconstructs holographic images by recording the interference pattern of light scattered by the specimen. This technique enables researchers to observe cells in their natural environment without the need for staining or labeling, preserving their integrity and minimizing artifacts. DHM offers real-time imaging, allowing dynamic monitoring of cellular processes such as migration, division, and interaction. Its non-invasive nature makes it ideal for studying live cells, providing valuable insights into fundamental biological mechanisms [69].

Combining Raman microscopy with DHM enables a comprehensive label-free and non-destructive method with real-time capabilities. The advantage of this combination lies in concurrently obtaining biochemical information from Raman spectroscopy along with morphological information and refractive index of the cell. Studies indicate that cells, throughout various cellular cycles, exhibit not only different biochemical behaviors but also varied morphologies [29,70]. While Raman microscopy provides precise information about the biochemistry governing the cell and cell status, the weak Raman signal necessitates considerable acquisition time for obtaining Raman images. Conversely, digital holographic microscopy can capture three-dimensional images of numerous cells simultaneously in a very short time (less than 1 s). Therefore, the combination of these two methods can not only provide a deeper understanding of cellular processes but also enhance precision and operational speed [71]. Additionally, digital holography can serve as a high-speed primary screening method, while Raman spectroscopy acts as a secondary method with high accuracy [72].

One of the most crucial cellular stages in studying anti-cancer drugs is apoptosis, or programmed cell death. Research indicates that the onset of apoptosis may coincide with significant biochemical changes such as the breakdown of proteins [73]. In addition to being observable in the Raman spectrum of the cell, these biochemical changes lead to alterations in cell morphology and mitochondrial network (volume reduction), which are observable with digital holographic microscopy. This morpho-molecular correlation not only aids in the better design of anticancer drugs but also provides a more accurate depiction of the cell’s status [74].

**Figure 4:**
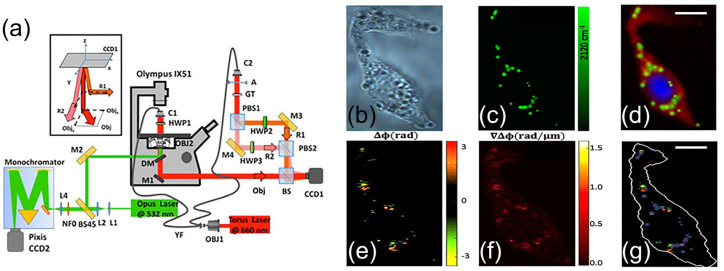
(**a**) Combined Raman and polarization-sensitive digital holographic imaging (PSDHI) experimental setup, (**b**) bright field image of the HepG2 cell, (**c**) Raman map of the C-D band signal, and (**d**) reconstructed false color Raman image using the DNA Raman bands at 2956 cm^−1^ and 785 cm^−1^ for the nucleus (blue signal), the protein bands at 2930 cm^−1^ and 1100 cm^−1^ for the cytosol (red signal) and C-D bands at 2120 cm^−1^ for the lipid droplets (green signal), (**e**) Phase difference; and (**f**) the corresponding phase difference gradient maps retrieved by PSDHI, (**g**) Merged image of the Raman map of the C-D band signals and the phase difference map by PSDHI maps, assessing the co-localization of the C-D signal of the lipid droplets and the state of polarization variation. Scale bar:10 μm. Reprinted (adapted) with permission from Ref. [26], Copyright 2023 Frontiers in Bioengineering and Biotechnology.

Different metabolisms governing healthy and cancerous cells lead to biochemical and morphological differences that can be useful for high-precision differentiation between healthy and cancerous cells. For example, according to the Warburg effect, cancer cells exhibit up to 10 times greater glucose uptake compared to their healthy counterparts. Research shows that glucose taken up by the cell can be stored as fatty acids and cholesterol esters. Cholesterol esters, due to their ordered and regular structures, are highly sensitive to light polarization. Mangini et al. utilized the combination of Raman microscopy and polarization-sensitive digital holographic imaging (PSDHI) to demonstrate that lipid droplets act as reservoirs for cholesterol esters and fatty acids. Figure 4a shows the experimental setup for combined Raman microscopy and PSDHI. Figure 4c shows the false color Raman image of the carbon-deuterium vibrational band at 2120 cm^−1^, indicating higher glucose uptake compared to healthy cells. Furthermore, the results of PSDHI in Figure 4e indicate that lipid droplets in cancer cells have a higher degree of birefringence with a good correlation with Raman images. These findings could be highly beneficial for rapidly distinguishing between healthy and cancerous cells even at speeds close to video rate [26].

### 2.4. Correlative Raman and Mass Spectroscopy Imaging

Mass spectrometry imaging (MSI) is a sensitive, label-free imaging analysis technique with a wide detection range. MSI is not limited to a specific molecule but can simultaneously obtain the spatial distribution, relative content, and structural information of multiple biomolecules in biological tissues or cells. Since a large number of cellular constituents are Raman-active to some degree, a superposition of spectral information from proteins, lipids, and nucleic acids is represented in the Raman spectra of biological tissues [75]. Applying MSI as a correlated method that collects specific molecular information (molecular weight of the ion divided by its charge, *m*/*z*) can visualize the spatial distribution of molecules with single-molecule detection limits [76].

**Figure 5:**
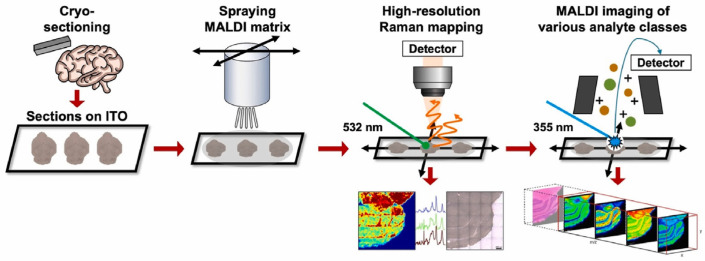
Experimental workflow for correlative Raman–MALDI imaging. A fresh-frozen tissue section is cryo-sectioned and thaw-mounted onto a conductive indium tin oxide (ITO) microscopy slide. This is followed by MALDI matrix application by spraying 1,5-diaminonaphthalene (DAN) onto the tissue section. The prepared tissue section is first utilized for Raman imaging, and then the same sample is subjected to MALDI MSI measurement. Reprinted (adapted) with permission from Ref. [82], Copyright 2023 Elsevier.

MSI allows for mapping the distribution of molecules within tissue samples by generating molecular maps of cancerous tissues; it can identify spatially specific molecular signatures associated with tumor regions. MSI employs various ionization techniques like matrix-assisted laser desorption/ionization (MALDI), secondary ion mass spectrometry (SIMS), and desorption electrospray ionization (DESI) [77]. Figure 5 shows the workflow for correlative Raman–MALDI imaging. Briefly, in MALDI-MSI, a sample is coated with a matrix and irradiated with a laser to desorb and ionize molecules, while SIMS uses a focused ion beam to sputter secondary ions from the sample. DESI creates charged droplets to interact with the sample and produce ions. These ions are then separated based on their mass-to-charge ratio and detected. A mass spectrum, containing all the mass signals of the desorbed compounds, is acquired for each point of the raster. Subsequently, a dataset comprising an ordered array of mass spectra is generated, with each spectrum representing the local molecular composition at known x, y coordinates. Ultimately, an image can be generated for each of the mass signals detected throughout the section. In particular, the intensities of individual *m*/*z* values in each spectrum, corresponding to the molecular masses of specific compounds, can be extracted to produce images of the areas within the tissue where that particular molecule was located. This process is akin to digital imaging in photography, where each image is composed of an ordered array of thousands of pixels [78].

MSI and Raman imaging can be combined to overcome the limitations of one technique and complement it by the advantages of the other. Molecules that do not ionize efficiently may produce a strong vibrational signature or vice versa; thus, by combining both methods, chemical coverage is expanded [77]. Combining Raman imaging and MALDI-MSI offered enhanced characterization of epithelial differentiation and deeper insights into dysplastic alterations in larynx carcinoma [79]. 

Despite all advantages of combined Raman–mass spectroscopy imaging, there are some difficulties for producing correlated images [76]. Principally, Raman imaging has a far better resolution compared to MALDI-MSI [79]. To overcome the difference in spatial resolution, a group has developed correlated Raman imaging and MALDI-MSI to study the main regions of proliferation in three-dimensional (3D) cell cultures for cancer studies [80]. On the other hand, both techniques have their own data format that makes it difficult to compare the final images. A group converted Raman imaging datasets collected from mouse brain tissue and then visualized them with commonly used MSI software tools [81].

Another challenge in correlating these modalities is the difference in sample preparation protocols. However, recent advancements have demonstrated seamless integration of Raman imaging and MALDI MSI for various tissue types from a single sample [82]. As a result, correlating Raman imaging and MSI can complement histology and pathology because the diagnosis can be supported by objective criteria with the spectral information.

## 3. Summary and Future Perspective

The combined power of Raman microscopy with confocal fluorescence microscopy (CFM), atomic force microscopy (AFM), digital holography microscopy (DHM), and mass spectroscopy imaging (MSI) offers a comprehensive toolkit for cancer researchers, clinicians, and drug developers. Each of these correlative approaches brings unique strengths to the table, providing a multidimensional view of cancer biology that transcends the limitations of individual techniques. Raman microscopy’s ability to non-invasively and label-freely characterize the biochemical fingerprint of cancer cells has been significantly enhanced by correlating it with other imaging modalities. CFM provides spatial context and allows for the study of cellular processes in real-time, while the development of dual-tagging molecules further strengthens this synergy. Correlative Raman–AFM has emerged as a powerful tool for analyzing the biomechanical properties of cancer cells, which play a crucial role in tumor progression and metastasis. This combination enables the identification of cancer-specific alterations in cell stiffness, elasticity, and adhesion and allows for the detailed study of subcellular structures and drug-induced changes in cell morphology and biomechanics. DHM’s ability to capture label-free, three-dimensional images of cells in real-time, coupled with Raman spectroscopy’s biochemical insights, offers a unique platform for studying dynamic cellular processes. This combination enables the identification of morpho-molecular correlations in cancer cells, providing valuable information for drug development and the study of cellular cycles. Finally, MSI adds another dimension to cancer research by offering spatial distribution and relative content information of multiple biomolecules in biological tissues. The integration of Raman microscopy with MSI allows for the study of a broader range of molecules and expands the chemical coverage in cancer analysis.

Despite the numerous advantages, challenges remain in the correlative use of these techniques. Differences in spatial resolution can make it difficult to correlate features observed with one modality to those observed with another. For example, Raman microscopy typically offers higher chemical specificity but lower spatial resolution compared to fluorescence microscopy. Additionally, sample preparation protocols for each technique may vary, requiring researchers to develop specialized workflows or compromise on the quality of information obtained from one modality to optimize for another. For instance, some techniques require tissue fixation or staining, which can alter the biochemical composition of the sample and potentially compromise the information obtained from Raman spectroscopy. Conversely, other techniques may necessitate the use of specialized buffers or mounting media that are not optimal for Raman analysis.

Challenges remain in translating these powerful research tools into clinical practice. The high cost and complexity of some techniques may limit their accessibility in certain settings. Additionally, the integration of multiple imaging modalities into a cohesive workflow can be time-consuming and require significant technical expertise. Furthermore, data analysis pipelines for correlative microscopy are still under development, and the lack of standardized data formats can pose challenges for data sharing and collaboration. However, ongoing efforts to develop cost-effective and user-friendly platforms hold promise for the widespread adoption of correlative microscopy in cancer diagnosis and treatment monitoring [83].

The integration of artificial intelligence (AI) into correlative Raman microscopy is poised to revolutionize cancer research by addressing current limitations and unlocking new possibilities. AI algorithms, for instance, can analyze vast and complex datasets from multiple imaging modalities (e.g., Raman, AFM, and DHM), identifying subtle correlations between biochemical changes, such as altered lipid profiles detected by Raman, and biomechanical properties, like cell stiffness measured by AFM [84]. This could lead to the discovery of novel cancer biomarkers and therapeutic targets. Moreover, AI-powered image analysis tools can automate the detection and classification of cancer cells based on their unique Raman spectral signatures, potentially improving the accuracy and efficiency of cancer diagnosis [85]. By harnessing the power of AI, correlative Raman microscopy can transcend its current limitations and pave the way for a new era of personalized cancer medicine.

In conclusion, the correlative use of Raman microscopy with other microscopy techniques represents a paradigm shift in cancer research. It empowers researchers with unprecedented capabilities to unravel the complex mechanisms underlying cancer, facilitating the development of more effective diagnostic tools, personalized therapies, and ultimately, improved patient outcomes. As technology continues to evolve and our understanding of cancer deepens, the convergence of these diverse imaging modalities will undoubtedly play a pivotal role in shaping the future of cancer research and transforming the way we diagnose and treat this devastating disease.
